# Collections and Common Sense

**Published:** 1916-03

**Authors:** 


### Collections and Common Sense.

There appears to be a prevailing idea among physicians, as well as among the laity, that doctors should give their services to every person who applies to them, regardless of whether the persons pay for such service or not. In fact, so strong has this sentiment become that many physicians claim that half of the practice is done without any expectation of remuneration.

There is no doubt that this condition exists, and looking at the matter lightly it may not impress one as a very bad state of affairs, but, viewed more critically in the light of common sense and reason, it impresses one as a condition which should not be tolerated for a moment.

Why should a physician give his services gratuitously any more than a lawyer or a teacher or any other professional man? It is merely a matter of custom, and it is largely the fault of the profession that this custom prevails.

Before the day of city and county physicians and charity hospitals this practice was commendable and humanitarian, but now that every county and city of any size has its health officers who are paid salaries out of the public treasury to look after persons who are not able to pay for the services of a physician, it should be no longer tolerated by medical men.

There are persons who never pay a doctor's bill but who go the rounds from one physician to another relating their ills and taking the doctor's time until they feel they are no longer welcome when they pass along to the next unsuspecting physician. Such people should be exposed. It is not the right spirit to encourage in American people. If one is not able to pay for the services of a private physician, he should be told to apply to the city or county physician for help.

When one takes into consideration the expense of a medical education, the cost of a well-equipped office, the high price of instruments and the fact that the physician must always present a good personal appearance; that he must supply himself with good literature and take post-graduate work, there is reason enough why he should be paid well for the service he renders.

The custom of rendering service for nothing should be stopped; medical men should place the proper value on their labors and insist upon getting it.

Some physicians flatter themselves that rendering service for nothing gives them added standing—a kind of a place apart. There is no doubt that it gives them standing with a certain class, but if one wishes to know the true financial standing of physicians as a class let him have a talk with the bankers in his town, and he will find that their condition is never too good. Every physician should adopt some good, sane method of collecting his bills, and should insist upon being remunerated for his services. The man who is too indolent and lazy to keep up his collections really does not deserve the sympathy of any one. Such sympathy should be reserved for his unfortunate family, which is by his negligence made to suffer the consequence of his business methods.

---

VOLUNTARY INERTIA.—"Your husband, madam," said the doctor as gravely as he could, "is suffering from voluntary inertia."

"Oh, my poor Henry!" sobbed the good woman, "and here I've been telling him he was just lazy."—*Fun.*

